# Engagement of health and social care employers in professional regulatory fitness to practise – missed regulatory and organisational opportunities?

**DOI:** 10.1186/s12913-025-12343-2

**Published:** 2025-02-15

**Authors:** Louise M. Wallace, Mari Greenfield

**Affiliations:** Faculty of Wellbeing, Education and Language Studies, Open University, Walton Hall, Milton Keynes, UK

**Keywords:** Professionals, Regulation, Communication, Fitness to Practise, Employers, Liaison, Misconduct

## Abstract

**Background:**

Health and social care professional regulators are organisations that ensure their registrants have the correct qualifications and experience to practise in their profession. There are 13 statutory regulators in the United Kingdom (UK) and 29 voluntary accredited registers. Referrals of serious concerns about registrants’ Fitness to Practise (FtP) are investigated by regulators, and can lead to a public hearing. Employers may refer their employee to the regulator, and provide evidence about the concerns about their practice. Communication between the regulator and employer is central to ensure fitness to practise procedures are timely and effective, contributing to patient safety and to the improvement of health and social care professional and organisational practices.

**Methods:**

In this mixed-methods research, semi-structured interviews were conducted with 25 participants who held senior management roles in health and social care organisations in the UK and were responsible for communicating with professional regulators. Descriptive statistics were produced relating to participants’ roles and organisations, and qualitative data was analysed using Template Analysis methodology.

**Results:**

Four themes relating to communication between employers and regulators were identified: Process of regulatory investigation, Point of contact with employers, Local level/informal resolutions, and Organisational learning. Employers frequently described the processes as protracted and stressful for all concerned, and communication with regulators as sporadic and unidirectional during investigations. This style of communication hampered organisational learning from Fitness to Practise cases. Regulators’ employer liaison officers, where they existed, improved communications.

**Conclusions:**

Fitness to Practise processes create the opportunities for not only the determination of whether an individual professional is fit to practise and the supportive measures that might need to be taken if they continue to practice, but also for health and social care organisations to prevent occurrence and re-occurrence of misconduct, thereby improving their services. Regulators’ communication patterns resulted in these employers’ organisational opportunities being missed. It may also lead to over-referral thereby leading to burden on employers, registrants and regulators. Improvements in communication by regulators such as via dedicated employer liaison functions could help organisations access these opportunities as well as promote the objectives of regulators to uphold trust in regulation.

**Supplementary Information:**

The online version contains supplementary material available at 10.1186/s12913-025-12343-2.

## Background

More than 2 million health and care professionals in the United Kingdom (UK) are required by law to be on the register of their professional regulator. Health and social care regulators are organisations that ensure members have the correct qualifications and are fit to practise to the required professional standards in their profession. There are 13 statutory health and care regulators in the UK. Fitness to practise (FtP) refers to the qualifications, health, character, and competence of a professional to practise. Where a concern is raised to the regulator about a registrant, the regulator may carry out an investigation. Concerns may be raised by anyone, and most often includes the public and employers. Regulators contribute to patient safety by ensuring professional standards are upheld and by doing so, they aim to maintain public confidence in the professions [1].

The ‘[FtP] proceedings are generally inquisitorial’ ([2], p.15) and involve legal processes which are widely recognised as being significantly stressful for both the public [3, 4] and registrants involved in this process [3–7] even occasionally leading to registrants’ suicide [8].

This paper comes from a research project independent of and focussed on the 13 statutory UK regulators which investigated the experiences of those who raised concerns, and those who were called as witnesses in FtP processes, including patients and service users, their friends and family members, who had alleged harm [9]. It also included colleagues who had witnessed, alleged or themselves experienced incidents of harm. It included senior managers of registrants’ employing organisations, where they may be called to provide evidence about any internal investigations, and previous health, competence, or disciplinary proceedings.

As different professions are regulated by different regulators, employers may work with multiple regulators, each with somewhat different professional standards, regulations and operational procedures. See Supplementary Materials 1. The purpose of professional regulation is to protect the public, uphold professional standards and maintain public confidence in the profession [1]. To achieve these purposes the professional regulator maintains a register of those who are appropriately qualified (and in some professions, indemnified) and have a sufficient knowledge of English. To remain on the register, the registrant must adhere to the profession’s standards. Their fitness to practise may be compromised by reason of ill health, incompetence, misconduct, or because they are convicted of specific crimes, among other concerns. These matters are investigated by the regulator to identify whether they pose a risk to patients’ safety and confidence in the profession. If the matter is serious, it may be adjudicated by a hearing before a panel appointed by (but independent of) the regulator. The onus is on the regulator to prove their case, and the registrant has the right of a defense. Hearings are usually conducted in public, and outcomes are published. As established by legal precedent, judgments on the facts are made on the civil standard of proof, i.e. the balance of probabilities, while matters of the seriousness of the breach are a matter of judgement. The outcomes, while varying between regulators according to their rules, include there being no case to answer, warnings or reprimands, restrictions on practice or being struck off the register [10].

Most professional regulators have no powers of inspection but rely on people and organisations raising concerns about a health and social care professional registered with them. Concerns are most often raised by the public, registrants and by employers, as well as other agencies such as the police. Some professions have standards that require them to report concerns about colleagues. Health and care services regulated by the organisational regulator may report professionals to their professional regulator [11]. Regulators rely on these sources to raise concerns when necessary for public protection, and not to make referrals which cannot be substantiated or are insufficiently serious, for example. Regulators’ investigations may rely on evidence (such as healthcare records, reports, e-mail and social media data, witness statements, and disciplinary proceedings) being submitted by the employer. The employer may be required to enable employees to make witness statements, and to attend a hearing to be cross examined. While under investigation, the registrant may be restricted by the regulator in their practice, which may need to be managed by the employer (10, Chapters 43,44). The regulator therefore has an interest in providing and maintaining effective communications with the employing organisations.

Employers have a reciprocal interest in ensuring there is effective communication between themselves and the regulators. Employers rely on regulators to ensure that those they employ are appropriately trained, qualified and suitable individuals to carry out the health or social care tasks of the profession. Health and social care organisations have an interest in learning from complaints and adverse events, to improve safety and outcomes for those in their care [12], and to ensure they raise only the most appropriate concerns. The body with oversight of nine of the UK's health regulators and the social work regulator for England, the Professional Standards Authority (see Supplementary Materials 1 for details), recommends there are opportunities from the FtP process to contribute to “upstream” regulatory improvements in the practices of employers to prevent FtP concerns arising in future [1]. When an employees’ fitness to practise is investigated, learning could occur both from the employers’ own investigations, and from the FtP processes where new evidence may be presented, thereby improving their organisational practices. Of the 13 regulators relevant to this study, three had an employer liaison function in 2022 (see Supplementary Materials 1).

In this research, we investigated the ways in which employers supported registrants, colleague witnesses and public witnesses during FtP processes (Wallace L, Ryan S, Searle R, Hughes G, Sorbie A, Ryan-Blackwell G, et al: Witness to Harm-Holding to Account. Improving patient, family and colleague witnesses’ experiences of Fitness to Practise proceedings: A mixed methods study, in press). As part of this work we asked about communication between employers and regulators. From this data we formulated our research question: ‘How do employers perceive communication with regulators, and what impact does this have on employers’ ability to engage and learn from FtP?’.

## Methods

We sought to understand the perspectives of health and social care employers about FtP. To gather rich data about participants’ experiences of being responsible within their organisation for all aspects of engaging with the regulators’ FtP processes we employed semi-structured interviews, using an interview guide developed for this project. (See Supplementary Materials 2 for interview guide). The Open University’s Human Research Ethics Committee granted approval for the study (OU HREC No. 4058).

### Recruitment

To meet the inclusion criteria, participants had to hold a senior role within a health or social care organisation in the UK which employed registered health and care professionals. They had to hold responsibility within the organisation for information at all stages of the FtP process about the registrant employee’s fitness to practise. The source of the concern to the regulator could be from any source, including their patients or service users.

Participants were recruited in three ways. First, contact was made with organisations where senior staff had previously been involved in another research project [13] who were invited to nominate a participant who fitted the inclusion criteria. Second, health and social care professionals in senior positions in health and social care organisations who were panel members from the funders’ national research committee for health and care services research were also invited to take part. Larger corporate employers in the pharmacy and optical sectors were approached directly. Snowball recruitment strategies were then used: individuals who were recruited via nomination or direct invitation were asked to let other suitable candidates within their own organisation or their peers in similar roles within other organisations know about the research [14].

### Data collection

Semi-structured interviews were offered to 27 people and 25 people took part. Participants either gave written consent prior to the interview via an online link or gave verbal consent at the beginning of their interview, which was recorded. All interviews were carried out by the lead author over Microsoft Teams software, audio recorded and transcribed by professional transcribers. Interviews lasted between 30–60 min.

### Analysis

Descriptive statistics were collated relating to participants’ job titles, organisational type, registrant types and numbers of registrants the participant was responsible for, and the approximate number of new concerns being raised to and managed by regulators per annum. Transcripts were then transferred to NVivo and anonymised, with job titles and organisational information removed, as well as names and locations. Answers to the open ended questions were analysed using Template Analysis – a specialist form of Thematic Analysis in which data is coded iteratively to produce and refine a template, which is then re-applied to all transcripts [15]. The template produced consisted of a range of themes, one of which was ‘Communication with regulators’, which is reported on in this paper.

## Findings

### Quantitative findings

Interviews were carried out with 25 participants. The employing organisation was based in at least one of all four of the countries of the UK. Some reflected on more than one role they had held, as in previous organisations they had worked for, or had experience within their role in working with more than one regulator, providing a total of 32 experiences of 11 of the regulators. The distribution of organisations that participants represented is shown in Table 1 below.

**Table 1:** Interviewees’ organisational background and FtP responsibilities

Broad organisation of employers (n = 25)	Specific organisational type		Registrant employee professions involved in FtP
National Heath Service (NHS) (*n* = 12)	NHS Board	3	Doctors Psychologists Dentists Healthcare scientists Optometrists Pharmacists Physician associates Allied health professionals Osteopaths
	NHS General hospital and community services Trust	3	
	Mental health and community services Trust	6	
Private health and /or social care provider (*n* = 6), with some operating in one or more areas of provision	Private mental health/learning disability care	5	Social workers Nurses Allied health professionals Social care staff
	Private residential care for older people	5	
	Private residential care for children	3	
Corporate pharmacy business (*n* = 3)	3		Pharmacists
Local authority social services (*n* = 4)	4		Social workers Allied health professionals

There was a wide variation in the number of ongoing FtP investigations of individual employees (referred to hereafter as cases) that interviewees were responsible for. This variation largely reflected the variation in the size of the relevant workforce, as shown in Table 2 below.

**Table 2:** Number of FtP cases participants oversaw

Organisational type	Range of new FtP cases annually
NHS	1–30
Private health and /or social care provider	1–72
Corporate businesses	6–12
Local authority social services	1–3

### Qualitative analysis

Within the topic of Communication with Regulators, five themes were identified. These were: Process of regulatory investigation, Point of contact with employers, Local level resolutions, Support from regulator to witnesses, and Organisational learning. Each theme is discussed below.

#### Theme 1 - Process of investigation by the regulator

Communication between the employing organisation and the regulator was perceived by interviewees as being sporadic and demanding and operating in one direction only – regulators contacted employers to ask for certain information, but regulators did not routinely update employers.

‘I just feel they’ve all gone in a hole and I’m chasing and then what happens is out of the blue you get some call that says oh you’re going to be part of something, we now need 50 million bits of paper and we’re going to interview you.’ (Interview 19)

‘I've had to chase [regulator] for an update on this. They haven't come willingly to me. It's important to be providing those updates rather than have to kind of chase it.’ (Interview 21)

‘There was long periods where we had no contact, then suddenly a rush, we need your statements within a day or whatever. Then there’s a long gap between that and what comes back is not what you [wrote]. And again when it was sent back for checking, it was you need to send it back to us within a really short timeframe.’ (Interview 6)

This style of communication was felt to impose a significant burden on the employer, and specifically on the interviewees (who were responsible for responding on behalf of the organisation). Similarly, this was described by interviewees as imposing a burden on colleague witnesses who may be called to give evidence as part of the regulator’s case which it was unrealistic to expect them to bear alongside their role as a healthcare practitioner (HCP).

‘It’s got to be realistic when there’s people who are completing this work for us [who] are clinical. Sometimes we get things and they want a return within five working days.’ (Interview 9)

Interviewees also frequently commented that the lack of communication meant the process of investigation was not transparent to the employer. Sometimes organisations were not made aware of what was being investigated, or who else had been approached for evidence. Whilst this may on some occasions have been the necessary result of the regulatory processes involved in FtP, it also posed some difficulties, in that it limited the organisation’s ability to support colleague witnesses if they did not know they were involved:

‘We don’t know when someone’s been asked… because of the way that that request is made, it doesn’t come from the {regulator} to us as an organisation. It goes direct to the individual registrant that might be asked to give witness. So we don’t always get that opportunity to be able to talk to the person and support them because it sometimes will come out of the blue.’ (Interview 1)

Several interviewees commented that they had witnessed cases from the same regulator which appeared to them to be very similar, but where different processes or outcomes occurred, but the differences were not explained by the regulator to the employer. In one case, allegations were made by a family member about neglectful care against two nurses who worked together, where all the evidence was identical from the interviewee’s perspective:

‘Investigation for one of the {registrants}was closed down, within about a month, but the other one has remained open, and yet the allegations against both of them, the evidence provided is exactly the same… And the challenge for us as the employer is that we see that this one person has been absolutely exonerated, no case to answer, thank you very much, that’s all done, challenging for her but, you know, it’s done, and yet we have a colleague on the other hand who we cannot understand what is different between the two. Other than the fact that we’ve got different investigating and case management investigation teams who are clearly treating this slightly differently.’ (Interview 1)

Another participant had overseen two very similar cases in the same period, with very different outcomes, and that he had no route to make sense of this, further damaging trust.

‘I’ve referred two [registrants] to the [regulator] for inappropriate sexual advances on junior members of staff. And interestingly the approach taken by the [regulator] in both cases was completely different. In one they took the [registrant] for an FtP and in the other they threw it out at triage and the two cases were on the surface exactly the same… I still haven’t really got a satisfactory answer as to why the approaches were different.’ (Interview 3)

Interviewees also reported that the lack of transparency during investigations and the time it took damaged the trust from employers in regulators and potentially impacted the support for the employee to be at work and consequently the safety of services they provide, as evidenced in the quote below.

‘By the time they started asking us questions about this [registrant], she'd been off sick when the issue first came up, she'd… been back in work for a year by the time [regulator] came back to us. Well, really. You've gotta hope that we're right and that she's fine to be back at work. Haven't you? {Because} that's a year down track?’ (Interview 18)

#### Theme 2 – Point of contact with employers

Interviewees had a range of experiences in terms of whether there was a point of contact with the regulator. In 10 of the 13 UK regulators there was no system of communication in place other than for receiving concerns and obtaining information from the employer. All 13 regulators on our advisory group confirmed the presence or absence of employer liaison functions. One regulator had a named person who the employer could contact about FtP cases, and two regulators had a liaison function which extended beyond discussing cases to include, for example, training sessions for staff, and continuing professional development requirements. As some interviewees were responsible for individuals registered with more than one regulator, they were able to comment on the differences in communication they experienced because of the different systems. Where there was a named individual point of contact, this was always seen as better than a system without this.

‘I think having some kind of a link system for [organisations] where you’ve got a name to contact where you build your relationship with them [is useful]. Because with the [regulator] case we didn’t have anybody to go to say we haven’t heard anything… if we’d had that link I would have been able to pick up the phone to them and say can you find out what’s happening here so I could support the staff in that space.’ (Interview 6)

Regular meetings with the regulator were preferred over ad hoc meetings, and regular meetings were seen as easier to arrange when there was a defined liaison role.

‘One thing I do always welcome is meeting [with the liaison person] regularly… You can talk through concerns and once you’ve got that relationship with them, you can pick up the phone and say what do you think? Because sometimes you can go through [a different regulator’s] employer advice line, and sometimes I don’t agree with their advice, because I don’t know who you get on the other end of the phone.’ (Interview 9)

Regular meetings also facilitated the regular review of cases. In some cases, employers and regulators had very different lists of which cases were active, and which had been closed. Regular reviews helped avoid this situation.

‘In January we had 260 referrals that were not closed to either ourselves or the [regulator]… We’ve been laboriously going through each of our lists. Some of them match, some of them don’t. I’ve been in post five months now… we’re nearly at the stage where we have a shared list between us and the [regulator]. So we have reduced our caseload at the moment to 165… we’ve reduced a hundred in that period.’ (Interview 13)

#### Theme 3 – Local level resolution

Interviewees had a range of different viewpoints about mismatches between local level resolution of some issues and FtP concerns. There was a general view that the current way in which communications about FtP happen hampers good local resolution.

‘The {regulator} [is] shifting their emphasis about what to refer…even if it technically reaches threshold, it could be managed locally. I think that’s really welcome… we need to move to a more restorative approach… And I have a really good relationship with the (officer) now about what they think needs to come in and it’s really, you know, it’s sexual crime or gross dishonesty. But in respect of the clinical performance, very little progresses in it to an FTP or a clinical performance, so I think we need to learn that… and not refer {registrants} in on a clinical performance if we can manage it ourselves.’ (Interview 3)

For other participants the lack of clarity about what concerns to raise led to concerns about over-referring, which was unanimously seen as an avoidable harm for registrants, colleague witnesses, and for already over-stretched regulators.

‘We need more guidance on what to refer and what not to refer, because if a member of staff has been involved in poor performance… then should they really be referred? I don't think so, as a responsible employer… in the past I've had conversations with people at fitness to practise where they say you should tell them about everything and they then become a pseudo human resources department.’ (Interview 24)

However, there was a recognition that local resolution processes could be inappropriate or impossible, especially when the registrant was employed on a temporary contract or in a locum position.

‘Because this wasn’t an employed [registrant], it was a locum, there was no disciplinary process…had that have been an employed [registrant] they would have been contacted as part of that internal investigation. Because that person wasn’t employed, we still took witness statements from them, and then said no thank you, we don’t want you to come and work for us again, and reported them to the regulator.’ (Interview 15)

‘I’ve seen an increase in locum ones that we’ve referred rather than employees, which are tricky, because we don’t know their background.’ (Interview 17)

The challenges of local resolution led to several interviewees commenting that they made a significant number of referrals to the regulator for temporary staff:

‘And of those, and I think this is an important one for us, of that average about 50% will be agency staff that we refer.’ (Interview 1)

Taken together, participants’ responses gave the sense that local resolution and FtP processes sometimes contradicted each other rather than working harmoniously, and that a finer grained and individualised approach by regulators as to what should be referred to FtP could be beneficial. Greater co-ordination between employers and regulators might also reduce case failure and indirectly support employers to keep their employees operating safely.

#### Theme 4 – Support from regulator to witnesses

Two interviewees described how in sexual misconduct cases the handling of the case by the regulator resulted in women who had alleged sexual assault withdrawing their evidence. In one case the interviewee reported that this was as a result of the trade union representative’s aggressive questioning during the FtP Hearing, which was not challenged by the regulator-appointed tribunal:

‘She got a very difficult time from the trade union who were representing.’ (Interview 4).

In the absence of a formal role or comprehensive guidance for either the regulator or the employer in providing support to witnesses and guidance to the panel on managing appropriate cross examination, vulnerable witnesses may feel unsupported during adversarial style cross examination in FtP Hearings.

In a second case the person withdrew during the investigation:

‘We had a sexual misconduct case with a young girl [colloquialism, the interviewee is speaking about a young adult woman]… we referred this case, and the investigator contacted her, she felt that she was on trial because of the line of questioning… she didn’t have any obligation to be a witness in the end, she dropped out and so then they don’t really have a case then.’ (Interview 17)

Poor support from the regulator to the witness could make the case collapse, and had implications for local level resolutions as it could jeopardise any further contractual or disciplinary action by the employer.

#### Theme 5 – Organisational learning from FtP

Interviewees were asked about organisational learning from FtP cases. Effective organisational learning is the responsibility of the organisation, but may depend on the information from the regulators. Amongst those factors that the regulator could control, the transactional style of communication between the regulators’ legal team and the employer was described by interviewees as hampering organisational learning from FtP cases.

‘I ended up writing a statement for [regulators’] solicitors. That dragged on probably for about two years. And when that was finally dealt with, despite me providing the statements and assuming that at some point I would be called to justify those statements and be questioned on those statements, the matter was dealt with and seemingly dismissed. I don't know because I never had a formal outcome to it.’ (Interview 20)

One important facet of organisational learning which some participants mentioned was learning whether they were raising appropriate concerns. Where the regulator had a liaison function which included regular contact with the same person, the ability to have an informal discussion about whether a registrant should be referred was seen as invaluable.

‘[I make an] initial phone call to, [FtP officer’s name, and say], what do you think? And then if he says yes, well, it's obviously then a formal referral. And if it's, if there's no, then it stays there… I just I go to him so I can chew the fat really and see what [FtP officer] thinks. (Interview 24).

Conversely, where no individual relationship existed, participants reported having to deal with significant bureaucracy to get an answer about whether a referral was even appropriate.

‘You'll get someone other [a person you have not had contact with before] {at the} end of the phone. They'll say ‘I'm not sure, just send in the application’. You’re like, ‘Oh my God, can you make a decision? You know it's gonna take me a bit to fill in that application and then send you everything’. And it would be great if there was just an online form that you could fill in for your service that said, ‘I've got a query here. This is what we've done so far, is this fitness to practise?’ (Interview 25)

Interviewees were also asked about factors which were within their organisation’s control. This included questions about what the organisation was learning from the FtP processes and the outcome of each individual case, and what structures supported the gathering and implementation of this learning. A minority of interviewees reported organisational structures which allowed for systematic and routine reporting within the organisation of new or resolved cases. Effective learning was more likely to be reported by participants when new and resolved FtP cases were a standing item on a Board-level agenda. However, in most cases any reporting of FtP cases happened in an ad hoc way, or the information was held at a level lower than the Board. When questioned, many participants reflected that this represented a missed opportunity for organisational learning and improvement.

Interviewer: ‘Is there any learning from what’s in the [FtP] database?

I would probably say not sufficient enough, no.

Interviewer: OK. Do you think it potentially could be used for learning purposes?

I absolutely do think it could be used; in fact, there’s probably an opportunity to do a wee bit more of that across [organisations].’ (Interview 5)

In general, FtP processes appeared to occupy an adjunct position within organisational learning and improvement structures, rather than being an integral part of organisational improvement. One factor influencing this could be the amount of time FtP processes take to get to an outcome, which is frequently several years. This was seen as a barrier to effective organisational learning by some participants, as practices had often changed by the time the FtP case had been heard. The factors the employer appeared to be able to control were therefore in reality dependent upon regulator-controlled processes, which made it challenging for the employing organisation to introduce structures to incorporate FtP learning.

‘There is often such a timeline between the incident [and] the FTP process concluding at any stage, that often in that we’ve lost the momentum around the learning.’ (Interview 1)

As can be seen in Table 2, the number of FtP cases organisations were involved with varied significantly, with some overseeing one new FtP concern a year, whilst others oversaw up to 72 new cases per year. Some participants said that the low number of cases their organisation dealt with were a barrier to organisational learning.

‘The numbers are relatively very low. You’re talking very much single figures in my experience, so not a huge number’ (Interview 11)

## Discussion

From this research, the most striking effect of the current system of communication between regulators and organisations employing health and social care registrants is such that employers report being hampered in understanding when to refer employees to their regulator, resulting in potential over-referral. Employers also report being sometimes unsure about how to contribute relevant evidence, and their role in supporting witness engagement. This could have three important outcomes.

First, the regulators may receive either too many or too few concerns from employers. However, from our respondents it seems that lack of clarity can lead them to err on the side of caution and to over refer. If concerns are raised that are inappropriate, resources will be wasted on unnecessary FtP investigations which are stressful for all involved. This will also lengthen the timescales of all investigations, with the potential to undermine the three purposes of regulation referred to in our introduction.

As shown in theme 5, a second potential outcome from poor communication by regulators to employers is that organisational learning from FtP cases may not be as effective as it could be. This could reduce the opportunities for prevention of occurrence or reoccurrence. It can also mean employers are missing opportunities to refine their referral thresholds and to dovetail their FtP referral with local investigations they would already undertake as the employer. This could lead to more inappropriate cases going forward.

Lastly, current communication practices also result in damage to employers’ trust in regulators and regulatory processes [16–18]. In our research we found that employers did not always trust the outcome of FtP investigations (see theme 1). In the absence of two-way communication, such incidents have consequences for whether organisational learning from FtP processes is seen by employers as warranted if they also have concerns about the quality of FtP investigations and hearings.

Employers also did not always trust the regulator to provide appropriate support to colleague witnesses. An example is the collapse of a sexual misconduct hearing described in theme 3 which arguably could have been prevented had the regulator prepared and supported the witness. As regulators provide regular training to panel members, their guidance could include when it is appropriate for the tribunal chair to intervene to protect the harmed witness from hostile questioning.

Current communication practices during FtP investigations create a significant burden, both for interviewees, and for the organisations they represented. Raising a concern and providing information on a regulator’s timescales therefore represents a significant investment on the part of the organisation. If this investment sees no return in terms of future organisational learning and improvement, there is a significant missed opportunity, with no instrumental benefit. Improvements to both employers’ internal systems for discussing FtP cases and subsequent learning within their organisation, and current communications between regulators and employers could offer improvements to this situation.

Improvements to the employers’ internal reporting of and learning from FtP cases could involve, for example, an annual report which compares this year’s figures to last year’s, which sections of the organisation FtP referrals have been located in, and whether each FtP case relates to other organisational procedures such as those concerned with adverse events, never events, near misses, and complaints by patients and service users. It could also be related to human resource policies on whistleblowing, staff grievances, recruitment procedures and contextual issues such as staff turnover and the use of temporary staff. Even for organisations which have few FtP cases, this model could be implementable, following the model of ‘never events’ [19]. Although these events are meant to never happen, it is a requirement to report on them. Adoption of this model would assist in improving organisational learning.

Improving communication between regulators and employers about regulation, including FtP processes would also assist employers and professionals to engage with FtP, aiding the regulator to fully investigate and present cases. Whilst regulators may face restrictions in the details they can give about the evidence they have gathered due to regulatory procedures, more frequent updates about the anticipated timeline of a case is not subject to such limitations. This would also assist the employer in providing the evidence and support to witnesses essential to these cases, and for the employer to learn and implement preventive quality improvement and professional practice measures. Having a liaison function attributed to a named individual in both the regulatory body and the organisation, as happened for some of our interviewees, helps to ensure that appropriate cases are referred. Equally, being kept abreast of the scope and progress of FtP investigations would promote trust in the processes. This might also ensure that communication about the process of each investigation involves more two-way traffic, rather than the current system of regulators making what are perceived as urgent demands for evidence, and employers chasing regulators for updates.

## Conclusions

Transactional and sporadic communication between health and social care regulators and organisations which employ health and social care registrants creates several missed opportunities. Creating effective liaison functions and ensuring communication is two-way should become the new norm for professional regulators.

## Supplementary Information

Supplementary Material 1.

Supplementary Material 2.

## Data Availability

The datasets generated and/or analysed during the current study are not publicly available due to the potential identifiability of individual interviewees but are available from the corresponding author on reasonable request.

## Abbreviations

FtP: Fitness to Practise
GMC: General Medical Council
NHS: National Health Service
HCP: Healthcare professional
