# Supplementary material for: Engagement of health and social care employers in professional regulatory fitness to practise – missed regulatory and organisational opportunities?
Source: BMC Health Serv Res. 2025 Feb 15;25:255. doi: 10.1186/s12913-025-12343-2 (PMC11829395; doi:10.1186/s12913-025-12343-2)
Supplement: Supplementary file 1 — Supplementary Material 1. [file 12913_2025_12343_MOESM1_ESM.docx]

Supplementary Materials 1

UK Health and Social Care statutory regulators

| **Name** | **Has a formal liaison function** | **Interviewees had experience of managing the organisational processes related to their employees’ FtP concerns** |
| --- | --- | --- |
| General Chiropractic Council (GCC)^*^ |  |  |
| General Dental Council (GDC)* |  |  |
| General Medical Council (GMC)* | **√** | **√** |
| General Optical Council (GOC)* |  | **√** |
| General Osteopathic Council (GOsC)^*^ |  | **√** |
| General Pharmaceutical Council (GPhC)* |  | **√** |
| Health and Care Professions Council (HCPC)* |  | **√** |
| Northern Ireland Social Care Council (NISCC) |  |  |
| Nursing and Midwifery Council (NMC)* | **√** | **√** |
| Pharmaceutical Society of Northern Ireland (PSNI) |  | **√** |
| Scottish Social Services Council (SSSC) |  | **√** |
| Social Care Wales (SCW) |  | **√** |
| Social Work England (SWE)* | **√** | **√** |

Footnote_ The existence of an employer liaison function was obtained from our project’s regulator advisory group which had input from the 13 regulators.

^*^Denotes regulators within the ambit of the Professional Standards Authority .
